# Supplementary material for: Futility in TAVI: A scoping review of definitions, predictive criteria, and medical predictive models
Source: PLoS One. 2025 Jan 9;20(1):e0313399. doi: 10.1371/journal.pone.0313399 (PMC11717200; doi:10.1371/journal.pone.0313399)
Supplement: S1 Table — (PDF) [file pone.0313399.s001.pdf]

Supporting information

S1. PICO framework

**PIcO**			
**(P)opulation**	**(I)ntervention**	**(C)omparison**	**(O)utcome**
- Patients with Aortic Stenosis	- TAVI	- No intervention	- Futility
