# Supplementary material for: Futility in TAVI: A scoping review of definitions, predictive criteria, and medical predictive models
Source: PLoS One. 2025 Jan 9;20(1):e0313399. doi: 10.1371/journal.pone.0313399 (PMC11717200; doi:10.1371/journal.pone.0313399)
Supplement: S2 Table — (PDF) [file pone.0313399.s002.pdf]

Supporting information

S2. Concepts and Medical Subject Headings (Mesh)

	Transcatheter Aortic Replacement	Futility	Risk assessment
Free Terms	Tavi<br><br>TAVI<br><br>Transcatheter Aortic Replacement	Futility<br><br>Medical futility	Benefice- risk assessment<br><br>Risk assessment<br><br>Predicting model<br><br>Patient outcome assessment
MeSH (PubMed)<br><br>(MEDLINE ovide)	"Transcatheter Aortic Valve Replacement"[Mesh]	("Medical Futility/ethics"[Mesh]) OR "Medical Futility"[Mesh]	((("Clinical Relevance"[Mesh]) OR "Risk Assessment/methods"[Mesh]) OR "Outcome Assessment, Health Care"[Mesh]) OR "Risk Assessment"[Mesh]
CINHAL Descriptors<br><br>(CINHAL)	(MH "Transcatheter Aortic Valve Replacement")	(MH "Medical Futility")	(MH "Risk Assessment")
Embase Descriptors<br><br>(Embase)	('transcatheter aortic valve implantation'/exp OR 'transcatheter aortic valve implantation')	0	'stratification'
(Cochrane Libary)		"futility"	"risk benefit assessment" "risk assessment"
