# Supplementary material for: Futility in TAVI: A scoping review of definitions, predictive criteria, and medical predictive models
Source: PLoS One. 2025 Jan 9;20(1):e0313399. doi: 10.1371/journal.pone.0313399 (PMC11717200; doi:10.1371/journal.pone.0313399)
Supplement: S3 Table — (PDF) [file pone.0313399.s003.pdf]

# Supporting information

**S3. Search equations**

| **Databases** | **Search Equations** | Results |
|---|---|---|
| **CINAHL** | (MH "Transcatheter Aortic Valve Replacement" or TAVI or TAVI) AND (MH "Medical Futility" or futility) | 36 articles |
| **PUBMED** | 1st research: (02.02.24) ("Transcatheter Aortic Valve Replacement"[MeSH Terms] OR "tavi"[Title/Abstract] OR "TAVI"[Title/Abstract] OR "transcatheter aortic valve replacement"[Title/Abstract]) AND ("medical futility/ethics"[MeSH Terms] OR "Medical Futility"[MeSH Terms] OR "futility"[Title/Abstract] OR "utility"[Title/Abstract]) AND ("Clinical Relevance"[MeSH Terms] OR "risk assessment/methods"[MeSH Terms] OR "outcome assessment, health care"[MeSH Terms] OR "Risk Assessment"[MeSH Terms] OR "risk stratification"[Title/Abstract] OR "Risk Assessment"[Title/Abstract] OR "predicting model"[Title/Abstract] OR decision making, shared[MeSH Terms]) | 130 articles |
| | 2nd research (10.08.24) ("Transcatheter Aortic Valve Replacement"[MeSH Terms] OR "tavi"[Title/Abstract] OR "TAVI"[Title/Abstract] OR "transcatheter aortic valve replacement"[Title/Abstract]) AND ("medical futility/ethics"[MeSH Terms] OR "Medical Futility"[MeSH Terms] OR "futility"[Title/Abstract] OR "utility"[Title/Abstract]) | 253 articles |

| | | |
|---|---|---|
| **Cochrane library, all databases** | transcatheter aortic valve replacement and futility<br><br>transcatheter aortic valve replacement and risk tools | 2 articles<br><br>1 article |
| **clinicalTrials.gov** | Aortic Valve Stenosis (disease)\| TAVI \(Transcatheter Aortic Valve Implantation\) \| Risk Stratification | 4 articles |
| **EMBASE (PICO TOOL)** | ('transcatheter aortic valve implantation'/exp OR 'transcatheter aortic valve implantation') AND futility | 73 articles |
