# Supplementary figures and images for: Futility in TAVI: A scoping review of definitions, predictive criteria, and medical predictive models
Source: PLoS One. 2025 Jan 9;20(1):e0313399. doi: 10.1371/journal.pone.0313399 (PMC11717200; doi:10.1371/journal.pone.0313399)

# Supporting information

## S4. Prisma Flow Chart (February 2024)

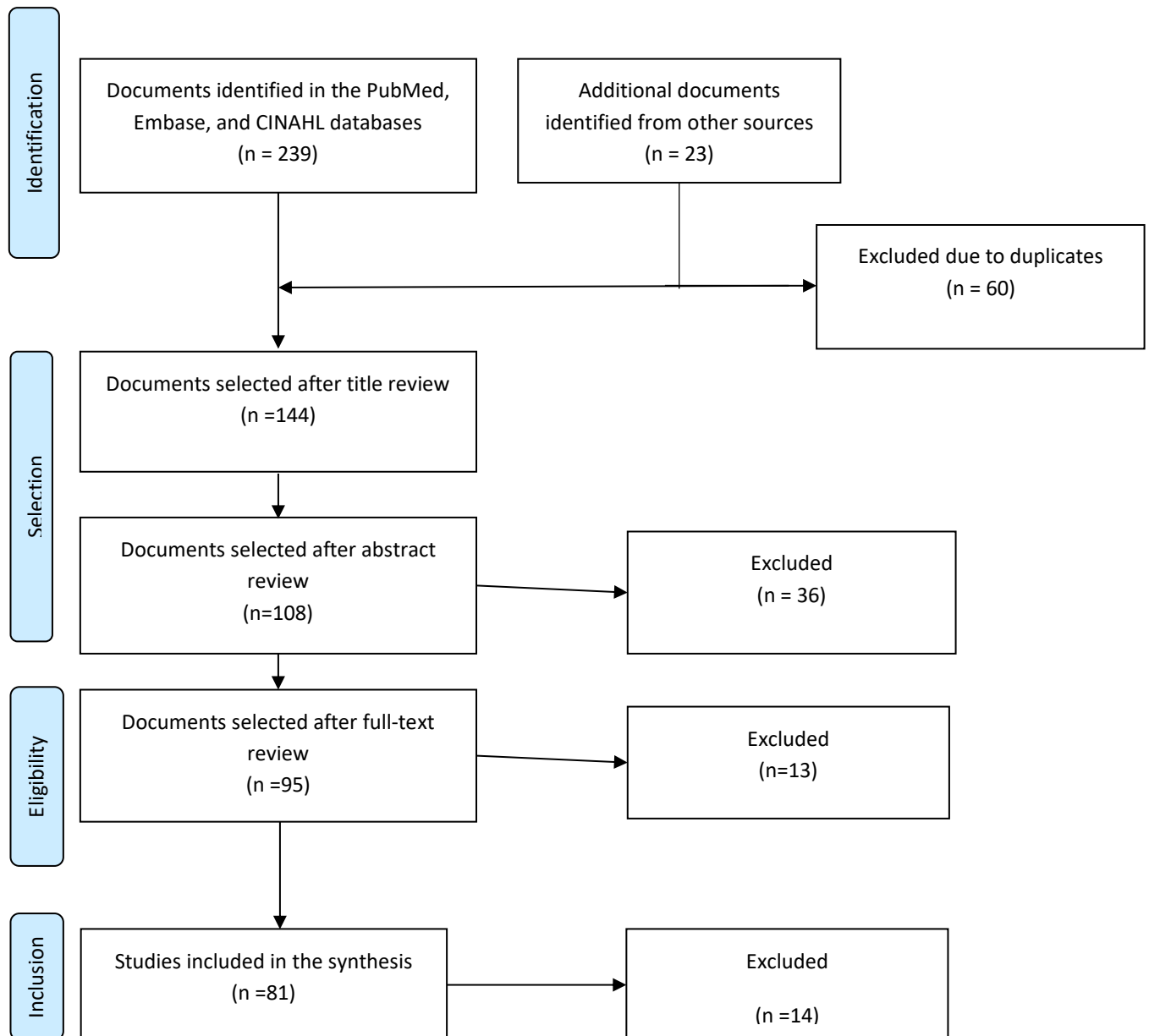

Supplement: S4 Table — (PDF) [file pone.0313399.s004.pdf]

# Supporting information

S5. 2<sup>nd</sup> search (08.24) on PubMed with wider equation.

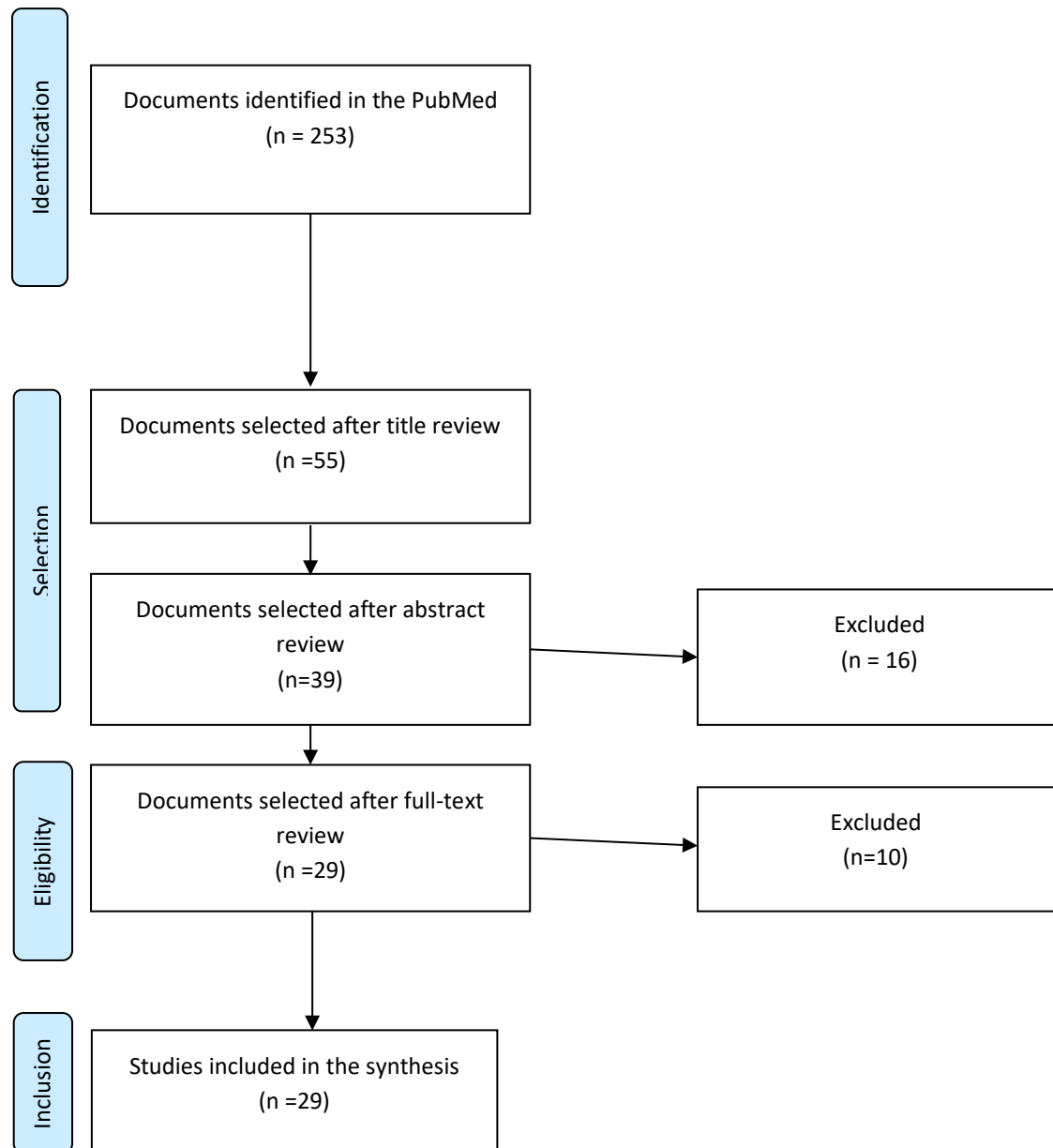

Supplement: S5 Table — (PDF) [file pone.0313399.s005.pdf]
