# Supplementary material for: Futility in TAVI: A scoping review of definitions, predictive criteria, and medical predictive models
Source: PLoS One. 2025 Jan 9;20(1):e0313399. doi: 10.1371/journal.pone.0313399 (PMC11717200; doi:10.1371/journal.pone.0313399)
Supplement: S6 Table — (PDF) [file pone.0313399.s006.pdf]

Supporting information

S6. Categories Used for Data Extraction

**Categories**	**Research question 1**	**Research question 2**	**Research question 3**	**Research question 4**
Reference	X	X	X	X
Date of publication	X	X	X	X
Country of publication	X	X	X	X
Study aims	X	X	X	X
Design	X	X	X	X
Sample size and description	X	X	X	X
Mean age of the sample	X	X	X	X
Instrument		X	X	X
Analysis	X	X	X	X
Definition of futility	X			
Themes / key findings on medical ethics	X	X	X	X
Themes / key findings on Comorbidities and TAVI and Futility		X	X	X
Tools stratification for TAVI intervention		X	X	X
Objective criteria of futility	X	X	X	X
Subjective criteria of futility	X	X	X	X
Reliability	X	X	X	X
Validity	X	X	X	X
Other key findings related to TAVI	X	X	X	X
Other key findings related to Futility	X	X	X	X
Shared Decision Making and TAVI	X			X
Frailty in TAVI	X	X	X	X
Comment on specificity to the research question	X	X		X
