# Supplementary material for: Futility in TAVI: A scoping review of definitions, predictive criteria, and medical predictive models
Source: PLoS One. 2025 Jan 9;20(1):e0313399. doi: 10.1371/journal.pone.0313399 (PMC11717200; doi:10.1371/journal.pone.0313399)
Supplement: S7 Table — (PDF) [file pone.0313399.s007.pdf]

# Supporting information

## S7. Included Studies Related to Research Questions

| Included studies | | Definition | | | | | | Predictors (cardiac) | | | | | | | | | Predictors (non cardiac) | | | | | | | | Fail to predict | | | | | | | | Medical predicting model | | | |
|---|---|---|---|---|---|---|---|---|---|---|---|---|---|---|---|---|---|---|---|---|---|---|---|---|---|---|---|---|---|---|---|---|---|---|---|---|
| | | Mortality <1y | Symptoms benefit | Qol improvement | Morbidity benefit | Patients’ goals, shared decisions | Financial ethics considerations | Previous Aorta or Cardiac damage | Low Flow Low Gradient (LFLG) | Valvulo arterial impedance | Valve disease other the aortic | Myocardial Function | Volumes and EF of the left atrium | Right Heart | Rhythms disorders | Biomarkers | Lung disease | Plasma volume Serum | Serum albumin level | Neutrophil to lymphocyte ratio | Frailty | Kidney disease | Demography | Comorbidities | LFLG | Cardiac Amyloid Pathology | Mitral regurgitation | Gradient adjusted vs. cardiac power | Spirometry | Cirrhotic patient | Previous hospitalizations | Platelet-to-lymphocyte ratio | Surgical model | Clinical predictive model | Machine learning | Heart Team |
| **Authors and date** | **study design** | | | | | | | | | | | | | | | | | | | | | | | | | | | | | | | | | | | |
| Adlbrecht et al. 2020 | Retrospective | | | | | | | | | | | | | | | | x | x | | | | | | | | | | | | | | | | | | |
| Afilalo et al. 2017 | Prospective | | | | | | | | | | | | | | | | | | | | x | | | | | | | | | | | | x | x | | |
| Agasthi, Ashraf H and al 2020 | Retrospective | | | | | | | | | | | x | | | | | | | | | | | | | | | | | | | | | | | x | |

| | | | | | | | | | | | | | | | | | | | | | | | | | | | | | | | | | | | | |
|---|---|---|---|---|---|---|---|---|---|---|---|---|---|---|---|---|---|---|---|---|---|---|---|---|---|---|---|---|---|---|---|---|---|---|---|---|
| Agasthi; Pujari SH and al 2020 | Prospective | | | | | | | | | | | | | | | | | | | | | | | | | | x | | | | | | | | | |
| Alhwiti et al. 2023 | Retrospective | x | | | | | | | | | | | | | | | | | | | | x | | | | | | | | | | | | x | |
| Allen et al. 2020 | Prospective | x | x | | | | | | | | | | | | | x | | | | | | | | | | | | | | | | | | | |
| Allen et al. 2017 | Retrospective | | | | | | | | | | | | | | | x | | | | | | | | | | | | | | | | | | | |
| Amat-santos et al. 2018 | Retrospective | | | | | | | | | | x | | | | | | | | | | | | | | | | x | | | | | | | | |
| Aquino et al. 2021 | Retrospective | | | | | | | | | | | | x | | | | | | | | | | | | | | | | | | | | | | |
| Arnold et al. 2013 | Retrospective | | | x | | | | | | | | | | | | | | | | | | | | | | | | | | | | | | | |
| Asgar et al. 2019 | Guidelines | | | | | x | | | | | | | | | | | | | | | | | | | | | | | | | | | | | |
| Beohar and al 2014 | Retrospective | x | | | | | | | | | | | | | | | | | | | | | | | | | | | | | | | | | |
| Bertschi et al. 2021 | Prospective | | | | | | | | | | | | | | | | | | | | x | | | | | | | | | | | | | | |
| Canatta et al. 2021 | Meta analysis | | | | | | | | | | | | | | | | | | | | | | | | | x | | | | | | | | | |
| Capodanno et al. 2014 | Retrospective | x | | | | | | | | | | | | | | | | | | | | | | | | | | | | | | | | x | | |
| Catalano et al. 2020 | Prospective | | | | | | | | | | | | | | | | | | | | | | | | | | | | | | | | | | | x |
| Coleywright et al. 2016 | Qualitative | | | | | x | | | | | | | | | | | | | | | | | | | | | | | | | | | | | |

| | | | | | | | | | | | | | | | | | | | | | | | | | | | | | | | | | | | | |
|---|---|---|---|---|---|---|---|---|---|---|---|---|---|---|---|---|---|---|---|---|---|---|---|---|---|---|---|---|---|---|---|---|---|---|---|---|
| Conrotto et al. 2017 | Retrospective | | | | | | | | | | | x | | | x | | | | | | | x | x | | | | | | | | | | | | | |
| Cook et al. 2019 | Retrospective | x | x | | x | | | | | | x | | | | | | x | | | | | | | | | | | | | | | | | | | |
| D'onofrio et al. 2014 | Retrospective | x | | | | | x | | | | | | | | | | | | | | | | | | | | | | | | | | | | | |
| Dagher et al. 2022 | Expert opinion | x | | | | | x | | | | | | | | | | | | | | | | | | | | | | | | | | | | | |
| Dagmar et al. 2019 | retrospective | | | | | | | | | | | | | | | | | | | | | | | | | | | | | | | | | | x | |
| Del Castillo et al. 2018 | Prospective | | | | | | | | | | | | | | | | | | | | | | | | | | | | | | | | | x | | |
| Diaz-Arocupita et al. 2023 | Review | | | | | | | | | | | | | | | x | | | | | | | | | | | | | | | | | | | | |
| Drakopoulou et al. 2021 | Retrospective | | | | | | | | | | x | | | | | | | | | | | | | | | | | | | | | | | | | |
| Erdogan et al. 2024 | Retrospective | | | | | | | | | | | | | | | | | | | | | | | | | | | | | | | | | | x | |
| Farshadmand e t al. 2022 | review | | | | | | | | | | | x | | | | | | | | | | | | | | | | | | | | | | | | |
| Fauchier et al. 2019 | Retrospective | | | | | | | | | | | | | | | | | | | | | | | | | | x | | | | | | | x | | x |
| Fennely and al 2018 | Case report | | | | | x | | | | | | | | | | | | | | | | | | | | | | | | | | | | | | |
| Freitas-Ferraz et al. 2020 | Retrospective | | | | | | | | x | | | | | | x | | | | | | | | | | | | | | | | | | | | | |
| Fukui et al. 2022 | Retrospective | | | | | | | | | | | | | x | | | | | | | | | | | | | | | | | | | | | | |

| | | | | | | | | | | | | | | | | | | | | | | | | | | | | | | | | | | | | |
|---|---|---|---|---|---|---|---|---|---|---|---|---|---|---|---|---|---|---|---|---|---|---|---|---|---|---|---|---|---|---|---|---|---|---|---|---|
| Fumugalli et al. 2021 | Prospective | | | | | | | | | | | | | | | | | | | | x | | | | | | | | | | | | | | | |
| Geisler et al. 2021 | Retrospective | x | | | | | x | | | | | | | | | | x | | | | x | | | | | | | | | | | | | | | x |
| Goitia et al. 2022 | Retrospective | | | | | | | | | | | | | x | | | | | | | | | | | | | | | | | | | | | | |
| Green et al. 2013 | Prospective | | | | | | | | | | | | | | | | | | | | x | | | | | | | | | | | | | | | |
| Gupta et al. 2020 | review | | | | | x | | | | | | | | | | | | | | | | | | | | | | | | | | | x | | | x |
| Hakgor et al. 2024 | prospective | | | | | | | | | | | | | x | | | | | | | | | | | | | | | | | | | | | | |
| Harbaoui et al . 2016 | Prospectuve | | | | | | | x | | | | | | | | | | | | | | | | | | | | | | | | | | | | |
| Hermiller Jr et al. 2016 | Retrospective | | | | | | | | | | | | | | | | x | | x | | x | | x | x | | | | | | | | | | | | |
| Hiesh et al. 2019 | Meta analysis | | | | | | | | | | | | | | | | | | x | | | | | | | | | | | | | | | | | |
| Higuchi et al. 2019 | Prospective | x | | | x | | | | | | x | x | | | | | x | | | | | | | | | | | | | | | | | x | | |
| Huang et al. 2023 | Review | | | | | | | | | | | | | | | | | | | | | | | | | | | | | | | | | | x | |
| Jacquemyn et al. 2024 | Review | | | | | | | | | | | | | | | | | | | | | | | | | | | | | | | | | | x | |
| Kappetein 2015 | expert opinion | | | | | | | | | | | | | | | | | | | | | | | | | | | | | | | | x | | | x |
| Khalil et al. 2018 | Retrospective | | | | | | | | | | | | | | | | | | | x | | | | | | | | | | | | | | | | |

| | | | | | | | | | | | | | | | | | | | | | | | | | | | | | | | | | | | | |
|---|---|---|---|---|---|---|---|---|---|---|---|---|---|---|---|---|---|---|---|---|---|---|---|---|---|---|---|---|---|---|---|---|---|---|---|---|
| Kjonas et al. 2021 | Prospective | x | | | | | | | | | x | x | | x | | | x | | | | x | x | | | | | | | | | | | | | | |
| Kreidieh et al. 2016 | Retrospective | | | | | | | | | | | | | | | | | | | | | | | | | | | | | | x | | | | | |
| Kuck et al. 2023 | cost analysis | | | | | | x | | | | | | | | | | | | | | | | | | | | | | | | | | | | | |
| Lak and al 2021 | observationnal study | | | | | | | | | | | | | | | | | | | | | | | | | | | | | x | | | | | | |
| Lantelme et al. 2018 | Expert opinion | | | | | x | x | | | | | | | | | | | | | | | | | | | | | | | | | | | | | |
| Lantelme et al. 2022 | Retrospective | | | | | | | | | | | | | | | | | | | | | | | x | | | | | | | | | | | | |
| Lauck et al. 2016 | Review | | x | | x | | | | | | | | | | | | | | | | | | | | | | | | | | | | | | | |
| Lauck et al. 2019 | Review | | | | | | | | | | | | | | | | | | | | | | | | | | | | | | | | | | | x |
| Liao et al. 2016 | Meta analysis | | | | | | | | | | | | | | | | x | | | | | | | | | | | | | | | | | | | |
| Lindman an al. 2014 | Review | x | | x | | x | | | | | | | | | | | | | | | | | | | | | | | | | | | | | | |
| Mack et al. 2017 | expert opinion | | | | | | | | | | | | | | | | | | | | x | | | | | | | | | | | | | | | |
| Martin et al. 2018 | Retrospective | | | | | | | | | | | | | | | | | | | | x | | | | | | | | | | | | | x | | |
| Maznyscka et al. 2020 | Retrospective | | | | | | | | | | | | | | | | | x | | | | | | | | | | | | | | | | | | |
| Medranda et al. | Retrospective | | | | | | | | | | | | | | x | | | | | | | | | | | | | | | | | | | | | |

| | | | | | | | | | | | | | | | | | | | | | | | | | | | | | | | | | | | | |
|---|---|---|---|---|---|---|---|---|---|---|---|---|---|---|---|---|---|---|---|---|---|---|---|---|---|---|---|---|---|---|---|---|---|---|---|---|
| Minha and al 2020 | Expert opinion | | | | | | x | | | | | | | | | | | | | | | | | | | | | | | | | | | | | |
| Molesh et al. 2021 | Retrospective | | x | x | | | | | | | | | | | | | | | | | | | | | x | | | | | | | | | | | |
| Mrack et al. 2016 | Case report | | | | | x | | | | | | | | | | | | | | | | | | | | | | | | | | | | | | |
| Murray et al. 2019 | Retrospective | | x | x | | | | | | | | | | | | | | | | | | | | | | | | | | | | | | | | |
| Nair et al. 2024 | retrospective | | | | | | | | | | | | | | | | | | | | | | | | | | | | | | x | | | | | |
| Navani et al. 2022 | Retrospective | | | | | | | | | | | | | | | | | | | | | | | | | | | | | | x | | | | | |
| Nitsche et al. 2020 | Retrospective | | | | | | | | | | | | | | | | | | | | | | | | | x | | | | | | | | | | |
| Nuis et al. 2020 | Prospective | | | x | | | | | | x | | | | | | | | | | | | | | | | | | | | | | | | | | |
| O’Leary et al. 2020 | Prospective | | | | | | | | | | | | | | | x | | | | | | | | | | | | | | | | | | | | |
| Okuno et al. 2021 | Retrospective | x | x | | | | | | x | | | | | | | | | | | | | | | | | | | | | | | x | | | | |
| Otto et al. 2017 | Guidelines | | | | | x | | | | | | | | | | | | | | | | | | | | | | | | | | | | | | |
| Papazoglou et al. 2024 | Review | | | | | | | | | | | | | | | | | x | | | | | | | | | | | | | | | | | | |
| Patel et al. 2022 | review | | | x | | | | | | | | | | | | | | | | | | | | | | | | | | | | | | | | |
| Pinar et al. 2022 | Cost analysis | | | | | | x | | | | | | | | | | | | | | | | | | | | | | | | | | | | | |

| | | | | | | | | | | | | | | | | | | | | | | | | | | | | | | | | | | | | |
|---|---|---|---|---|---|---|---|---|---|---|---|---|---|---|---|---|---|---|---|---|---|---|---|---|---|---|---|---|---|---|---|---|---|---|---|---|
| Pino et al. 2020 | Retrospective | | | | | | | | | | | | | | | | | | | | | | | | | | | | x | | | | | | | |
| Puri et al. 2015 | Review | x | | | | | x | | | | | | | | | | | | | | | | | | | | | | | | | | x | x | | |
| Rheude et al. 2017 | Retrospective | | | | | | | | | | | | | | | x | | | | | | | | | | | | | | | | | | | | |
| Rodrigues et al. 2018 | Expert opinion | | | | | | | | | | | | | | | x | | | | | | | | | | | | | | | | | | | | |
| Rodriguez-Gabella et al. 2018 | Prospective | | | | | | | | x | | | | | | | | | | | | | | | | | | | | | | | | | | | |
| Saji and al. 2016 | Prospective | | | | | | | | | | | | | | | | | | | | x | | | | | | | | | | | | | | | |
| Salizzoni et al. 2017 | Retrospective | x | | | | | | | | | | | | | | | | | | | | | | | | | | | | | | | | | | |
| Salihu et al. 2024 | Prospective | | | | | | | | | | | | | | | | | | | | | | | | | | | | | | | | | | x | x |
| Sevilla and al 2022 | cost analysis | | | | | | x | | | | | | | | | | | | | | | | | | | | | | | | | | | | | |
| Shimura et al. 2021 | Retrospective | | | | | | | | | | | | | | | | | | x | | | | | | | | | | | | | | | | | |
| Silaschi et al. 2015 | Comparative Analysis | | | | | | | | | | | | | | | | | | | | | | | | | | | | | | | | x | | | |
| Stein et al. 2024 | Prospective | | | | | | | | | | | | | | | | | | | | x | | | | | | | | | | | | | | | |
| Steinvil et al. 2018 | Retrospective | | | | | | | | | | | | | | | | | | | | x | | | | | | | | | | | | | | | |
| Tam and al 2018 | cost analysis | | | | | | x | | | | | | | | | | | | | | | | | | | | | | | | | | | | | |

| | | | | | | | | | | | | | | | | | | | | | | | | | | | | | | | | | | | | |
|---|---|---|---|---|---|---|---|---|---|---|---|---|---|---|---|---|---|---|---|---|---|---|---|---|---|---|---|---|---|---|---|---|---|---|---|---|
| Tang et al. 2020 | Retrospective | | | x | | | | | | | | | | | | | | | | | | | | x | | | | | | | | | | | | |
| Vahanian et al. 2021 | Guidelines | | | | | x | | | | | | | | | | | | | | | | | | | | | | | | | | | | | | x |
| Van Mourik et al. 2018 | Prospective | x | x | | | x | | | | | | | | | | | | | x | | | | | | | | | | | | | | | | | x |
| Wang et al. | Meta analysis | | | | | | | | | | | | | | | | | | | | | | | | | | | | | | | | x | | | |
| Wessler et al. 2020 | review | | | | | | | | | | | | | | | | | | | | | | | | | | | | | | | | | x | | |
| Xuan et al. 2019 | Retrospective | | x | | | | | | | | | | | | | | | | | | | | | | | | | | | | | | x | x | | |
| Zhou et al. 2023 | Prospective | | | | | | | x | | | | | | | | | | | | | | | | | | | | | | | | | | | | |
| Zusman et al. 2018 | Retrospective | x | x | | x | | | | | | | | | | | | | | | | | | | | | | | | | | | | | x | | |
