# Supplementary material for: Futility in TAVI: A scoping review of definitions, predictive criteria, and medical predictive models
Source: PLoS One. 2025 Jan 9;20(1):e0313399. doi: 10.1371/journal.pone.0313399 (PMC11717200; doi:10.1371/journal.pone.0313399)
Supplement: Attachment — Submitted filename: Response to rewiers Ferry and al..docx [file pone.0313399.s009.docx]

# Responses to the reviewers

Dear Dr De Rui,

Dear reviewers,

Thank you for your thorough review of our manuscript titled ***Futility in TAVI: A Scoping Review of Definitions, Predictive Criteria, and Medical Predictive Models.***

We appreciate the insightful comments and suggestions provided by the reviewers, which have greatly contributed to improving the quality of our work. In the following response letter, we address each of the points raised in detail. We have carefully considered all feedback and made the necessary revisions to the manuscript. Our responses are structured to correspond directly to the reviewers' comments for clarity and ease of reference.

We hope that our revisions meet your expectations and that the changes have enhanced the manuscript.

Thank you once again for the opportunity to submit the revised version, and we appreciate your time and consideration. We look forward to the possibility of seeing our work published in PLOS One.

Sincerely,

Charlie Ferry, PhD

# Responses to the queries

We thank the editors and reviewers for the careful and thoughtful analysis of our manuscript and answer the raised issues below:

| Reviews | Responses and actions |
| --- | --- |
| Journals requirements |  |
| 1. Please ensure that your manuscript meets PLOS ONE's style requirements, including those for file naming. | The first page has been modified to comply with the [guidelines](https://journals.plos.org/plosone/s/file?id=ba62/PLOSOne_formatting_sample_title_authors_affiliations.pdf).  The title has been updated, and the tables have been moved from the appendix into the manuscript. |
| 2. We noted in your submission details that a portion of your manuscript may have been presented or published elsewhere. | We apologize for any misunderstanding, which is likely our fault. However, we would like to clarify that no portion of this study has been published or presented elsewhere. The entire manuscript is original and has not been previously published. |
| 3. We note that your Data Availability Statement is currently as follows: [All relevant data are within the manuscript and its Supporting Information files.]  Please confirm at this time whether or not your submission contains all raw data required to replicate the results of your study. | Thank you for your inquiry. We confirm that this submission contains all the necessary data required to replicate the results of our study. As this article is a scoping review, all the data used have been sourced from existing literature, with no new calculations or modifications made. The search equations used to access the relevant articles are provided in Appendices 1, 2, 3, and 5. |
| 4. We note you have included a table to which you do not refer in the text of your manuscript. Please ensure that you refer to Table 3 in your text. | Thank you. Table 3 is referenced in the text within the chapter on the stratification tool. |
|  |  |
| Reviewer #1 |  |
| “For my taste, the text is a little bit too much of an enumeration, but probably that’s due to the type of article.” | Thank you for your feedback. We agree that the text might feel somewhat like an enumeration, which was indeed a challenge. However, this style is somewhat inherent to scoping reviews due to the nature of summarizing a wide range of sources. Nevertheless, we have made some adjustments to a few sentences to improve the flow and readability. |
| “Maybe just reduce the papers that you describe more in detail and concentrate more on the conclusions drawn from your findings” | Thank you for your insightful feedback. We understand the concern raised regarding the detailed description of the papers. However, the style of a scoping review necessitates an exhaustive overview of all relevant articles. Reducing and concentrating the information would alter the nature of the review, potentially shifting it towards a different type of review. We appreciate your understanding of the specific requirements of this methodology. |
|  |  |
| Reviewer #2 |  |
| Lines 70-79: These seem reasonable but I urge the authors to exercise caution when making recommendations or commenting on clinical application since this is a scoping review and the studies were not critically appraised. We can't speak with any kind of certainty as to findings, we can only be descriptive of what we're seeing in the literature (for the most part I think you have achieved this well, but I have flagged a few areas in the manuscript where language could be softened a bit). | Thank you for your valuable feedback. We have taken your suggestion into account and have softened the language in the results and discussion sections to better reflect the descriptive nature of the findings. |
| Line 82: Please consult PRISMA 2020 for Abstracts as there are a number of reporting items that could be improved (I know it's technically for systematic reviews but since there isn't currently one for scoping reviews many of the items are still relevant!). | We acknowledge that eligibility criteria and the name of the information source should align more closely with PRISMA 2020 for Abstracts. However, no formal assessment of bias was conducted, as the review aimed to map the literature. Additionally, we did not use a protocol registration. This is a valuable point, and we will certainly consider it for future reviews. |
| Line 90: Please note that PRISMA is a reporting guideline not a conduct guide - please reword this and consider consulting something like the JBI manual, Arksey & O'Malley, Levac, Munn or one of the other seminal scoping review guidance papers | This is an important distinction, thank you.  We rephrase the sentence like this:  “A scoping review was conducted by two researchers and reported in accordance with the PRISMA-ScR guidelines.” |
| Line 133: Please state your rationale for using PRISMA for Systematic reviews instead of PRISMA-ScR. | This is a mistake when rewriting the manuscript. We used Prisma for Scoping reviews following the steps on the [website](https://www.prisma-statement.org/scoping). |
| Lines 134-138: Should this be in the protocol/registration section? | You're correct. We have renamed this section to 'Protocol' accordingly. |
| Line 141: Please specify which of the 6 Cochrane Library databases were searched (or all of them), as well as platforms that Embase and Cochrane were searched on (Embase.com or Ovid for Embase, Wiley or Ovid for Cochrane). | We searched all Cochrane databases, and Embase was accessed via Embase.com using the PICO tool. |
| Line 144: Did the librarian write the search or did they only provide guidance?  If the former, have they been extended the offer of co-authorship if they aren't in the authorship list already, and if the latter, have you asked whether they would like to be acknowledged if they haven't been already? | Thank you for your comment. The librarian provided guidance during the initial stages of the search process but did not write the search strategy herself. We agree that it is important to acknowledge her contribution, and we will ensure she is properly credited in the acknowledgments section. |
| Line 145: Thank you for including your search strategies! This is excellent reporting best practice. When were these searches run?   I also wonder if including risk stratification in your PubMed search is unnecessarily reducing your results. Finally, depending on what platform you searched it on, your Embase search syntax looks incorrect (there should be field codes for the keywords and the /exp is in the wrong location for an Ovid search). | Thank you. We included the date (November 2023).  Thank you. It's true that some of our equations yielded smaller results. For Embase, we utilized their PICO tool, which we deemed sufficient at the time, though we acknowledge that a more refined equation could have been used. |
| Line 146: Please describe your cross-referencing methods in a bit more detail. | We reviewed the references of the articles we read in full and identified additional articles that appeared relevant to our research subject. These cross-referenced articles were then further evaluated for inclusion. This process is now described in the 'Search' section of the manuscript. |
| Line 168: Typically scoping reviews will provide a tabular summary of their results (often referred to as "Table 1") where they try their best to summarize the salient information from the various studies. You kind of do this with Table 3, and Appendix 6 but neither provide a broad overview of the literature in tabular form (e.g. citation, population, study or publication type, definition of futility used, predictors reported, tools reported). It might be worthwhile to look at other scoping review examples to see how others have attempted to represent this information (it's definitely challenging, especially with larger included study counts), as this specific table is often the strongest contribution that scoping reviews bring to the literature, as it allows readers to get a high-level overview of the literature study by study very quickly | Thank you for the feedback. Given the large number of articles included, it was indeed challenging to create a readable table that covers all the questions comprehensively. However, we provide a more detailed table that offers a clearer overview than Appendix 6, while keeping Table 3 as it is. |
| Lines 176-208: Great summary! | Thank you. |
| Lines 209-282: Consider softening some of the language in this section, since none of these studies were critically appraised. For example "Concurrent conditions like COPD, atrial fibrillation, or a lower Stroke volume Index *were reported* to significantly raise the risk of TAVI futility". Reviewing the manuscript with a critical eye for this overall may be helpful. | Thank you for your suggestion. We have revised some of the phrasing in this section to soften the language, except in cases where the results are explicitly stated by the original research teams in their articles. In those instances, we have accurately reported their conclusions as presented. However, we have taken special care to avoid making any definitive statements in the discussion and conclusion sections. |
| Lines 402-409: Please also include limitations with regards to the conduct/execution of this study that you are submitting. | We had “Conducting a scoping review is a challenging process. One of the methodological limitations of this article is that it represents the authors' first attempt at performing a scoping review. Despite our rigorous efforts to adhere to international guidelines, we acknowledge that certain improvements could be made with a more experienced perspective.” |
| Final thoughts: This is generally well conducted and has a few minor clarifications and revisions needed. The biggest weaknesses in my mind at this time are the potentially overly simplistic and/or restrictive searches and the lack of a summary table of results. | Thank you again for your thorough review. We agree that our search equations were likely a bit too simplistic, although we believe the results provide a comprehensive overview that effectively addresses the research questions. We have updated the summary table as suggested. While we do have additional results, particularly for questions 2 and 3, including them all would have made the table difficult to read given the large number of articles. |
