# Supplementary material for: Futility in TAVI: A scoping review of definitions, predictive criteria, and medical predictive models
Source: PLoS One. 2025 Jan 9;20(1):e0313399. doi: 10.1371/journal.pone.0313399 (PMC11717200; doi:10.1371/journal.pone.0313399)
Supplement: Attachment — Submitted filename: Response to reviewers Ferry and al..docx [file pone.0313399.s010.docx]

# Responses to the reviewers

Dear Dr De Rui,

Dear reviewers,

Thank you for your thorough review of our manuscript titled ***Futility in TAVI: A Scoping Review of Definitions, Predictive Criteria, and Medical Predictive Models.***

We appreciate the insightful comments and suggestions provided by the reviewers, which have greatly contributed to improving the quality of our work. In the following response letter, we address each of the points raised in detail. We have carefully considered all feedback and made the necessary revisions to the manuscript. Our responses are structured to correspond directly to the reviewers' comments for clarity and ease of reference.

We hope that our revisions meet your expectations and that the changes have enhanced the manuscript.

Thank you once again for the opportunity to submit the revised version, and we appreciate your time and consideration. We look forward to the possibility of seeing our work published in PLOS One.

Sincerely,

Charlie Ferry, PhD

# Responses to the queries

We thank the editors and reviewers for the careful and thoughtful analysis of our manuscript and answer the raised issues below:

| Reviews | Responses and actions |
| --- | --- |
| Journals requirements |  |
| Please review your reference list to ensure that it is complete and correct. If you have cited papers that have been retracted, please include the rationale for doing so in the manuscript text, or remove these references and replace them with relevant current references. | All the references have been checked and are available. |
| Reviewer #2 |  |
| Unfortunately, while some of the content I had asked for is present, it's not very well reported (yet!). I would encourage the team to re-review PRISMA-Abstracts, PRISMA-ScR, and PRISMA-Search for reporting, and make sure that each item (if applicable) is reported in the right place (e.g. information sources in the methods section of the abstract, for example). It may be helpful to review the actual statement papers themselves for examples of how such items should be reported and in what detail | Thank you for your valuable feedback. We have revised the abstract to include the information sources, as follows: "We identified 129 studies from five key sources: CINAHL, PUBMED, the Cochrane Library, ClinicalTrials.gov, and EMBASE. The literature search was conducted in two rounds—first in February 2024 and again in October 2024—using no restrictions on the year of publication or the language of the studies. Additional references were included through cross-referencing."  Additionally, we recognize that the summary table of results was not placed correctly in the previous version. It should, in fact, be included as Appendix 7 (previously Appendix 6), which compiles all data extracted from the reviewed articles. The synthesis has now been shifted to the end of the Results section for better clarity and coherence. Moreover, the entire article has been reviewed according to PRISMA checklist criteria to ensure transparency and adherence to guidelines. |
| Appendix 6 you could further simplify the table by eliminating the "related to research questions" column as its largely repetitive of the much more interesting broken out sections. Also the sub-column "Definition" in the larger "Definition" column seems unnecessary. | Thank you, Appendix 7 was simplified with the removed of the section “related to question” and the first column “definition”. |
| I think Table 3 needs a bit more contextualization in the methods section for the categories, as not all of them are immediately obvious to the reader | Thank you, I think you may refer to Table 2. For more contextualization we moved this Table in the Results section and added “First, we find multiple concepts in definition of futility in TAVI. Secondly, we find 3 categories of predictive criteria (cardiac, non-cardiac and tested criteria who were not predictive). Finally, different type of medical predictive models. |
| I also still feel that the addition of the "clinical relevance" component of your PubMed search is at odds with the other database searches and as mentioned before, unnecessarily excludes articles. I would encourage the team to conduct and update and leave off the clinical relevance part of the PubMed search and search it from inception (rather than limiting by date as you may do the other databases) so that all the searches are the same across the databases (TAVI + futility). Your review would be greatly strengthened as a result | Thank you for your insightful comments. In response to your suggestion, we have updated our PubMed search by removing all terms related to "clinical relevance" in the second round of research. As a result, 263 articles were identified, following the same screening process as before. This led to the inclusion of 15 new articles, which strengthened our review(appendix 6). Specifically, two new items were integrated into the results: "Previous Aorta or Cardiac Damage" as a cardiac predictor and "Platelet-to-Lymphocyte Ratio" as a non-predictive variable. This adjustment has enhanced the consistency across all database searches and improved the robustness of our findings |
